# Hypoparathyroidism and Avascular Necrosis of the Hip Joint: A Case Report

**DOI:** 10.1002/ccr3.73360

**Published:** 2026-08-17

**Authors:** Munirah Altaissan, Amani A. Al‐Rajhi, Fahmi Yousef Khan

**Affiliations:** ^1^ Clinical Department, College of Medicine, QU Health Qatar University Doha Qatar; ^2^ Internal Medicine Hamad General Hospital Doha Qatar

**Keywords:** case report, femur head necrosis, hip joint, hypoparathyroidism, osteonecrosis, parathyroid hormone

## Abstract

A 30‐year‐old man presented with left hip pain and was diagnosed with femoral head avascular necrosis associated with idiopathic hypoparathyroidism, hypocalcemia, and vitamin D deficiency. Treatment with calcium, calcitriol, analgesia, and physiotherapy improved symptoms. This case suggests hypoparathyroidism may play a role in the development of avascular necrosis.

## Key Clinical Message

This case highlights hypoparathyroidism as a potential contributor to avascular necrosis (AVN) and underscores the need for further research into the role of parathyroid hormone in AVN development.

## 1 Introduction

Avascular necrosis (AVN), also known as osteonecrosis, aseptic necrosis, or ischemic bone necrosis, is a musculoskeletal disorder characterized by the death of bone tissue secondary to compromised blood supply [1, 2, 3]. AVN most commonly affects the femoral head, knee, talus, and humeral head [2]. Although several etiologies have been associated with the development of osteonecrosis [2], the role of low parathyroid hormone (PTH) levels as a contributing factor remains poorly described in the literature. Here, we report a case of AVN of the hip in a 30‐year‐old male with hypoparathyroidism.

## 2 Case History/Examination

A 30‐year‐old previously healthy gentleman was admitted to our center with a 3‐day history of severe left hip pain, which had gradually worsened over the preceding 3 months. The pain was described as throbbing in nature, localized to the left hip and buttock, with radiation to the thigh. The pain was aggravated by movement and weight‐bearing and reached a severity of 10/10 on the Numerical Rating Scale of pain, rendering him unable to ambulate. He also reported a one‐year history of progressive bilateral hand stiffness, intermittent shortness of breath, and voice changes. His mood was low. He had a history of *Helicobacter pylori* infection 1 year prior and was treated with standard eradication therapy. He denied smoking, alcohol consumption, trauma, steroid use, or radiation exposure. He is not on any regular medications. There was no family history of metabolic or endocrine disorders.

On physical examination, the patient weighed 57 kg and measured 167 cm in height, corresponding to a body mass index (BMI) of 20.4 kg/m^2^. He appeared uncomfortable due to the left hip pain. Examination of the left hip revealed a restricted range of motion, particularly during flexion and internal/external rotation, but without visible swelling, erythema, or warmth. Straight leg raise and FABER tests were negative. The neurological examination of the lower limbs was unremarkable, with preserved power and sensation. There were no cutaneous manifestations.

## 3 Methods (Differential Diagnosis, Investigations, and Treatment)

The differentials for the hip pain were AVN, fracture, septic arthritis, inflammatory arthritis, and malignancy.

Initial laboratory investigations revealed leukocytosis (white blood cell count 12.5 × 10^3^/μL (4–10 × 10^3^/μL), with an absolute neutrophil count of 9.5 × 10^3^/μL (2–7 × 10^3^/μL)), elevated C‐reactive protein (13.1 mg/L (0–5 mg/L)), and normal serum electrolytes.

Biochemical analysis demonstrated hypocalcemia with an adjusted calcium concentration of 1.80 mmol/L (2.20–2.60 mmol/L), hyperphosphatemia (2.42 mmol/L (0.8–1.5 mmol/L)), and an inappropriately low PTH level (7 pg/mL (15–65 pg/mL)). 25‐hydroxyvitamin D concentration was 15 ng/mL (20–40 ng/mL), while thyroid‐stimulating hormone, free thyroxine (T4), and magnesium levels were within normal limits.

Urinary calcium excretion was low (1.0 mmol/24 h (2.5–7.5 mmol/24 h)), suggesting hypocalcemia due to hypoparathyroidism. Autoantibody testing for antinuclear antibody (ANA) was negative.

**FIGURE 1:**
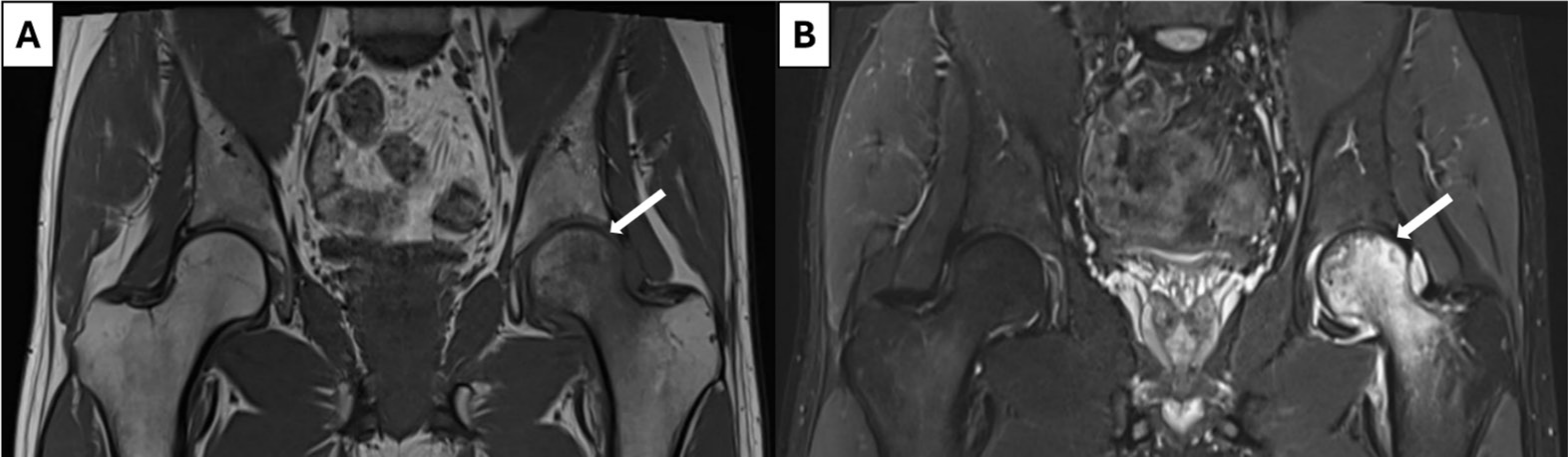
Magnetic resonance imaging (MRI) of the bilateral femoral heads. (A) T1‐weighted image demonstrating a limited subchondral linear‐shaped low signal intensity lesion in the left femoral head. (B) T2‐weighted fat‐saturated image showing corresponding heterogeneous signal changes and a possible “double‐line sign,” consistent with avascular necrosis.

Plain radiographs of the left hip revealed cortical irregularity and joint space narrowing. Bedside ultrasonography revealed no joint effusion. Magnetic resonance imaging (MRI) of the bilateral femoral heads revealed limited subchondral linear‐shaped low signal intensity on T1‐weighted images of the left femoral head, with corresponding heterogeneous signal changes and a possible “double‐line sign” on T2‐weighted sequences, which was consistent with avascular necrosis (Figure 1). Neck ultrasound revealed no detectable parathyroid enlargement or lesions.

A diagnosis of idiopathic hypoparathyroidism was made on the basis of the biochemical profile of hypocalcemia with low PTH in the absence of prior neck surgery, radiation, or evidence of autoimmune disease.

Established etiologies of AVN were assessed. The patient denied a history of hip trauma, corticosteroid use, excessive alcohol consumption, or radiation exposure. Autoimmune disease was considered unlikely given the absence of suggestive clinical features and a negative ANA test. Hemoglobinopathies were also considered unlikely based on the patient's personal and family history, complete blood count, and reticulocyte count. The patient had no known history of human immunodeficiency virus (HIV) infection, and a recent routine HIV screening test was negative.

The patient was initially managed with intravenous calcium infusion, followed by oral replacement therapy with calcitriol (0.5 mcg) twice a day and calcium carbonate (1500 mg) four times a day. Analgesia with ibuprofen and paracetamol was provided as needed.

## 4 Conclusion/Results

He was evaluated by orthopedic surgery for the suspected AVN and advised on conservative management with analgesia and physiotherapy. Rheumatology evaluation did not reveal any inflammatory arthropathy.

Over the course of hospitalization, the patient's serum calcium levels improved, and his hip pain gradually resolved. He regained mobility and was discharged home.

At the five‐month follow‐up, the patient reported feeling well with normal ambulation and no hip pain. His laboratory results revealed a low adjusted calcium level of 1.28 mmol/L (2.20–2.60 mmol/L) which, upon further questioning, was attributed to his noncompliance with medications. The patient was advised to take his medications. He was scheduled for follow‐up but was lost to follow‐up.

## 5 Discussion

We present the case of a previously healthy young man who developed AVN of the femur head in the setting of low PTH levels, raising the possibility of a contributory role of PTH in AVN development.

Osteonecrosis or AVN occurs because a reduction in the subchondral blood supply leads to hypoxia and necrosis of bone cells, which can cause irreversible joint damage and collapse [2, 3]. Therefore, high clinical suspicion and early diagnosis are crucial to prevent and minimize joint damage [2] and avoid the need for joint arthroplasty [4].

Few causes and associations have been linked to the development of osteonecrosis, which includes medications, such as glucocorticoids, alcohol consumption, trauma, and genetic disorders, such as sickle cell hemoglobinopathies [3]. However, some cases of AVN develop without an identifiable etiology and are therefore classified as idiopathic [5, 6].

**FIGURE 2:**
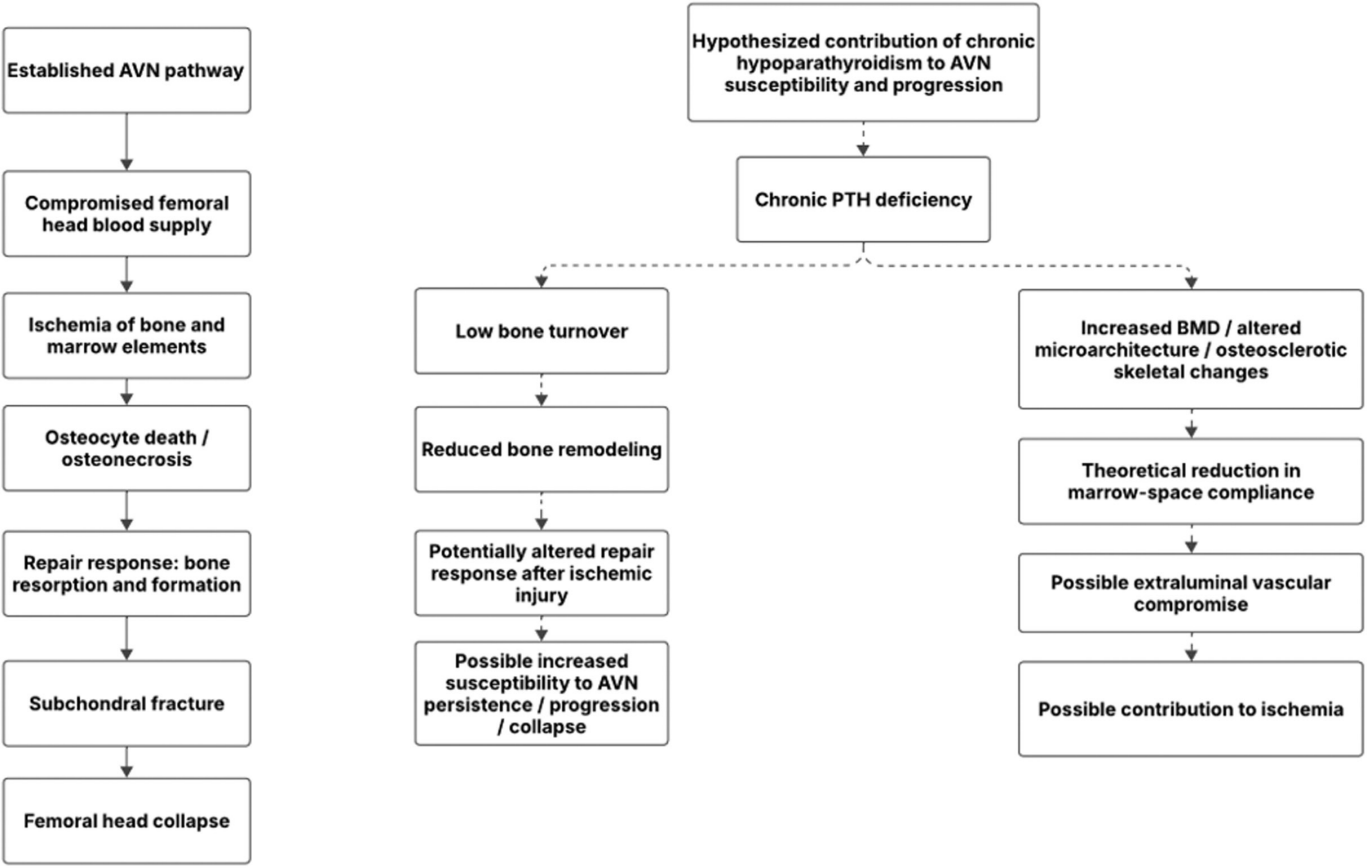
Proposed mechanisms linking chronic hypoparathyroidism to the progression of avascular necrosis (AVN). The left pathway summarizes the established pathophysiological sequence of avascular necrosis, in which compromised femoral head blood supply leads to ischemia of bone and marrow elements, osteocyte death, reparative bone resorption and formation, subchondral fracture, and eventual femoral head collapse. The right pathways illustrate hypothesized mechanisms by which chronic hypoparathyroidism may contribute to AVN susceptibility or progression. Chronic parathyroid hormone (PTH) deficiency is associated with low bone turnover and suppressed skeletal remodeling, which may theoretically reduce adaptive remodeling after ischemic injury and contribute to persistence or progression of osteonecrosis. In parallel, increased bone mineral density (BMD), altered skeletal microarchitecture, and osteosclerotic changes in chronic hypoparathyroidism may hypothetically reduce marrow‐space compliance and contribute to extraluminal vascular compromise, potentially worsening local ischemia. These proposed mechanisms remain hypothetical and are intended to illustrate biological plausibility rather than establish a direct causal relationship between chronic hypoparathyroidism and AVN. AVN, Avascular necrosis; PTH, Parathyroid hormone; BMD, Bone mineral density.

Hypoparathyroidism has a poorly defined association with avascular necrosis, and the available literature on non‐traumatic osteonecrosis does not establish true hypoparathyroidism as an independent cause of AVN [7, 8, 9]. However, a biologically plausible relationship may be proposed through the effects of chronic PTH deficiency on bone remodeling, skeletal microarchitecture. PTH is a key regulator of bone turnover, and chronic PTH deficiency is associated with reduced bone remodeling and increased bone mineral density (BMD) [10, 11]. Histomorphometric studies in hypoparathyroidism have demonstrated increased cancellous bone volume, increased trabecular width, increased cortical width, and markedly suppressed dynamic indices of bone formation [10]. Therefore, increased BMD in hypoparathyroidism should not be interpreted as normal bone quality or normal remodeling capacity, because chronic PTH deficiency is associated with altered skeletal microarchitecture and potentially abnormal bone strength [11]. In AVN, compromised femoral head blood supply leads to ischemia and death of bone and marrow cellular elements [8, 12]. Disease progression is influenced not only by the initial ischemic insult but also by the subsequent repair response, which involves both bone resorption and bone formation [8]. When this repair process is mechanically unfavorable, loss of structural integrity, subchondral fracture, and femoral head collapse may occur [8, 13]. In this context, chronic suppression of bone turnover in hypoparathyroidism may theoretically impair reparative remodeling after ischemic injury by reducing the capacity of necrotic or mechanically damaged bone to be removed and replaced [8, 10, 11]. This could contribute to lesion persistence, subchondral fracture, or progression toward femoral head collapse; however, this mechanism remains unproven in hypoparathyroidism‐associated AVN [8, 13]. A secondary theoretical pathway is the contribution of chronic PTH deficiency to ischemia through extraluminal, or extravascular compromise [8]. Extravascular compromise refers to reduced blood flow caused by compression of vessels from outside the vessel lumen rather than by intraluminal occlusion [8]. In osteonecrosis, this mechanism may occur when increased pressure within the rigid intraosseous compartment compromises perfusion of the femoral head vascular bed [8]. Because chronic hypoparathyroidism is associated with increased bone mass, altered skeletal microarchitecture, and radiographic osteosclerotic changes, a dense, low‐remodeling femoral head environment could theoretically reduce marrow‐space compliance or contribute to local osteosclerotic change [10, 11, 14]. This could theoretically increase susceptibility to pressure‐related compromise of intraosseous blood flow, but this proposed link is inferential and has not been directly demonstrated [8, 10, 11]. Direct evidence that hypoparathyroidism increases femoral head marrow pressure or directly causes AVN is lacking [7, 8]. Overall, hypoparathyroidism should be regarded as a possible contributing factor rather than a proven cause of AVN, with impaired reparative remodeling after ischemic injury and extraluminal vascular compromise representing two biologically plausible but unproven mechanisms [8, 10, 11]. This proposed mechanism is illustrated in Figure 2.

The available case‐based literature linking abnormal PTH physiology to femoral head osteonecrosis is concentrated primarily in pseudohypoparathyroidism and Albright hereditary osteodystrophy rather than in classic hypoparathyroidism [15, 16, 17, 18]. Because chronic hypoparathyroidism is associated with increased bone mass, altered skeletal microarchitecture, and radiographic osteosclerotic changes, a dense, low‐remodeling femoral head environment could theoretically reduce marrow‐space compliance or contribute to local osteosclerotic change. However, this proposed mechanism remains speculative and has not been directly demonstrated.

Valsaraj et al. stated that the exact cause of AVN in patients with pseudohypoparathyroidism is unknown, but suggested that it could occur as a serious complication of slipped capital femoral epiphysis rather than as a proven direct vascular consequence of abnormal PTH signaling [15]. Gertner et al. described normocalcaemic pseudohypoparathyroidism with elevated PTH resistance and imaging evidence of osteosclerosis and AVN of the femoral heads [16]. Lewington and El‐Hawary reported Legg‐Calvé‐Perthes disease in a patient with Albright hereditary osteodystrophy, providing another example of femoral‐head involvement in this disease spectrum [17]. Separately, Agarwal et al. reported pseudohypoparathyroidism type 1b as a rare cause of bilateral slipped capital femoral epiphysis, supporting the association between pseudohypoparathyroidism and endocrine‐related hip pathology [18]. Therefore, in the present case, hypoparathyroidism should be considered a possible contributing factor to impaired bone remodeling and structural repair rather than a proven direct cause of AVN.

A limitation of this report is the novelty of the proposed association between hypoparathyroidism and AVN. As limited literature currently exists to support this association, we suggest that further research is needed to reveal a possible association between hypoparathyroidism and the development of osteonecrosis.

In conclusion, the etiology of AVN is not well understood, and some idiopathic elements are worthy of further investigation. This case suggests a possible association between hypoparathyroidism and osteonecrosis. Larger studies are needed to clarify the role of PTH in the pathogenesis of AVN.

## Author Contributions

**Fahmi Yousef Khan:** conceptualization, investigation, writing – original draft, methodology, validation, visualization, writing – review and editing, project administration, supervision. **Munirah Altaissan:** conceptualization, investigation, writing – original draft, methodology, validation, visualization, writing – review and editing. **Amani A. Al‐Rajhi:** conceptualization, investigation, writing – original draft, methodology, validation, visualization, writing – review and editing.

## Funding

The authors have nothing to report.

## Ethics Statement

The authors have nothing to report.

## Consent

Written informed consent for publication of associated data was obtained from the patient himself.

## Conflicts of Interest

The authors declare no conflicts of interest.

## Data Availability

The data that support the findings of this study are available from the corresponding author upon reasonable request.
